# Overgrowth of the femoral neck after hip fractures in children

**DOI:** 10.1186/s13018-016-0387-9

**Published:** 2016-04-26

**Authors:** Feng-Chih Kuo, Shu-Jui Kuo, Jih-Yang Ko

**Affiliations:** Department of Orthopaedic Surgery, Kaohsiung Chang Gung Memorial Hospital, Chang Gung University College of Medicine, No. 123, Ta Pei Road, Niao Sung District, Kaohsiung City, 83301 Taiwan; Department of Orthopaedic Surgery, China Medical University Hospital, 2 Yude Road, Taichung City, 40447 Taiwan; Department of Orthopaedic Surgery, Xiamen Chang Gung Hospital, No. 123, Xiafei Road, Haicang District, Xiamen, Fujian China

**Keywords:** Overgrowth, Femoral neck fracture, Pediatric, Complications

## Abstract

**Background:**

Overgrowth after pediatric femoral shaft fractures is well documented; however, overgrowth of the femoral neck after hip fractures has not been especially reported previously. The purpose of this study was to evaluate the incidence and characteristics of femoral neck overgrowth after hip fractures in children.

**Methods:**

From January 1990 to December 2012, there were 30 consecutive patients with pediatric hip fractures. We retrospectively reviewed the medical record of all the patients, including age at injury, gender, injury mechanism, fracture type, methods of treatment, time to bony union, and complications. The functional outcome was evaluated by Ratliff’s criteria. The radiography of the pelvis was performed in controlled positions of abduction and external rotation. The length of the femoral neck was measured by two observers. The overgrowth of the femoral neck was defined as lengthening more than 3 mm in comparison with the uninjured hip.

**Results:**

At a mean follow-up of 4.9 years (range 2–8 years), 12 patients (40 %) had an overgrowth of the femoral neck. The average overgrowth of the femoral neck was 6.2 mm (range 3.2–8.5 mm). The patients with femoral neck overgrowth were younger (*p* = 0.0002), have lower rate of avascular necrosis of the femoral head (*p* = 0.0006), and have better functional outcome (*p* = 0.0026).

**Conclusions:**

Our results provide evidence that overgrowth of the femoral neck following hip fractures may occur in children and the overgrowth phenomenon in the femoral neck was a predictor of good outcomes after treatment.

## Background

Pediatric hip fractures are rare skeletal disorders compared with hip fractures in adult osteoporotic patients, accounting for less than 1 % of all pediatric fractures [1]. Accumulating evidence has demonstrated overgrowth after femoral shaft fractures in pediatric patients, with an average length of femoral shaft overgrowth ranging from 8.1 to 13.0 mm [2–6]. However, the overgrowth of the femoral neck after hip fractures has not been well documented [7].

We hypothesized that the overgrowth of the femoral neck might occur after hip fractures in children, as with the overgrowth of the femoral shaft after pediatric femoral shaft fractures. The purpose of this retrospective study was to analyze the incidence, the causing factors, and the influence of femoral neck overgrowth following hip fractures in children.

## Methods

Thirty pediatric patients (20 males and 10 females) with hip fractures were treated at our institute from January 1990 to December 2012. Patients older than 13 years, which were considered as adolescents and have less ability of proximal femoral overgrowth, were excluded in this study. The protocol for this retrospective study was approved by the Institutional Review Board of the hospital (Chang Gung Medical Foundation IRB No.: 98-3635B). Twenty-two patients had left hip fractures and 8 patients had right hip fractures. The mechanisms of injury varied, with falling from height being the most common factor (53.3 %). The other mechanism included motorcycle accident (20 %), bicycle accident (13.3 %), car accident (3.3 %), pedestrian-motor vehicle accident (3.3 %), basketball injury (3.3 %), and rope skipping injury (3.3 %). According to the Delbet system as popularized by Colonna [8], 5 were classified as Delbet type I (transepiphyseal separations with or without dislocation of the femoral head from the acetabulum), 17 were Delbet type II (transcervical fractures, displaced, and non-displaced), 8 were Delbet type III (cervicotrochanteric fractures, displaced, and non- displaced), and none were Delbet type IV (intertrochanteric fractures).

The treatment protocol for pediatric femoral neck fractures in our institute was described in the previous study [9]. Closed reduction and internal fixation were done for type I and displaced type II and type III fractures. An additional hip spica cast was applied when the patient was younger than 10 years old. If the surgeon failed to achieve anatomical reduction (<2 mm of displacement and <5° of angulation [10]) using closed method, open reduction and internal fixation were adapted.

The medical reports of all patients were reviewed, and data including age, gender, fracture type, method of treatment, time to bony union, age at last follow-up radiograph or evaluation, leg length discrepancy (LLD), and complications (avascular necrosis of the femoral head (AVN), nonunion, premature physeal closure, coxa vara, coxa valga, and infection) were recorded. According to the Ratliff criteria [1], the functional outcomes were classified as good, fair, and poor. AVN classifications were defined as type I (involvement of the whole head), type II (partial involvement of the head), and type III (an area of necrosis from the fracture line to the physis) osteonecrosis [1]. Nonunion was defined as implant breakage, loss of reduction, or persistence of a visible fracture line at a minimum of 6 months after the index procedure [10]. Premature physeal closure was defined as 50 % or more linear closure of the physis [11]. Coxa vara and coxa valga were defined as neck-shaft angles of less than 120° and more than 150°, respectively [12].

**Fig. 1:**
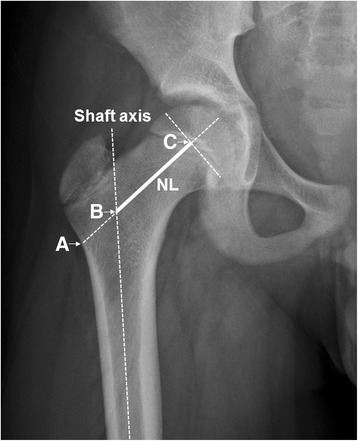
Scheme for the measurement of femoral neck length [13]. Femoral neck length (*NL*) was defined as the distance from the cross point (*B*) of the shaft axis and central axis of the femoral neck (*AC*) to the head center (*C*) measured along the central axis of the femoral neck. The neck-shaft angle is defined as the angle formed by the central axis and shaft axis. More than 3 mm of lengthening was defined as an overgrowth of the femoral neck (*B–C*)

The radiographic measurements were assessed by two investigators (FCK and SJK) using digital radiographs on a computer or conventional radiographs. Measurements were made from anteroposterior 1-m radiographs of the pelvis taken in a standardized fashion with the patient supine, placing the thighs and toes together, the beam centered on the midline, and the feet at 15°~30° internal rotation, which compensated for the normal anteversion of the femoral neck. Femoral neck length was defined as the distance from the shaft axis to the head center measured along the central axis of the femoral neck [13] (Fig. 1). The intraobserver and interobserver reliability of assessments of all radiographic measurements were assessed using intraclass correlation coefficients (ICCs), described by Konigsberg et al. [14]. The ICCs of the intraobserver and interobserver reliability of all measurements were greater than 0.85 (range 0.85–0.93) and considered to be reliable. We defined overgrowth of the femoral neck as lengthening by more than 3 mm in comparison with the uninjured hip.

### Statistical analysis

We compared differences in age using Mann-Whitney test. The functional outcome and the comparisons of causative parameters between overgrowth and non-overgrowth group were analyzed using Fisher’s exact test and chi-square test. A *p* value of ≤0.05 was considered to be statistically significant. All statistical comparisons were made using Statistical Package for Social Sciences (SPSS) software (version 20; Chicago, IL).

## Results

The mean age of the patients at the time of fracture was 8.2 years (range 1.5–12 years). The mean follow-up was 4.9 years (range 2–8 years). All patients had bone union at mean 11 weeks (range 4–20 weeks) after the injury. Using the Ratliff’s criteria [1], 16 patients had satisfactory outcomes (53 %). Fourteen patients had unsatisfactory outcomes (47 %) (11 fair and 3 poor). All patients underwent close reduction and internal fixation except one patient with type I fracture which was treated with open reduction and internal fixation because of failure of closed reduction. One patient with poor results was complicated with progressive coxa vara and underwent subtrochanteric valgus osteotomy 6 months after the initial operation. The other two patients with poor results had complications of Ratliff type I osteonecrosis, and they were kept under observation with nonsurgical treatment.

Overall, 11 patients had AVN (37 %), including 3 patients with transepiphyseal fractures, 7 patients with transcervical fractures, and 1 patient with a cervicotrochanteric fracture. All of them had unsatisfactory outcomes (8 fair and 3 poor). The other 19 patients without AVN had statistically significantly better outcomes (*p* < 0.001). Of the 11 patients who developed AVN, 4 were of Ratliff type I AVN with global involvement. There were 2 patients who developed Ratliff type II AVN and 5 patients were of Ratliff type III AVN. Nonsurgical treatment and observation were used for these patients. Six patients had premature physeal closure (20 %); however, there were no cases of nonunion or infections in this study.

**Fig. 2:**
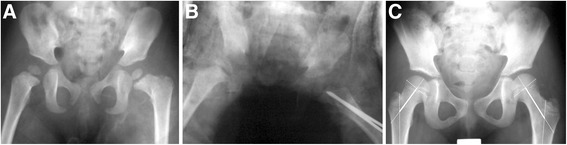
Representative radiographs of femoral neck overgrowth after a type III hip fracture. **a** A patient with a displaced type III fracture of the left femoral neck. **b** The two Steinmann pins did not penetrate the growth plate of the injured femoral neck after closed reduction and internal fixation. **c** Overgrowth (7.5 mm) of the left femoral neck was visible 4 years after injury

**Fig. 3:**
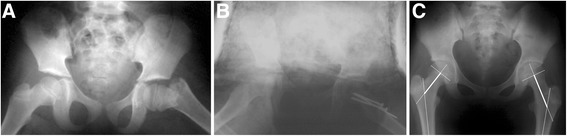
Representative radiographs of the femoral neck overgrowth after a type II femoral neck fracture. **a** A patient with a displaced type II fracture of the left femoral neck. **b** The two cannulated screws and one smooth k-wire did not pass through the proximal femoral growth plate after closed reduction and internal fixation. **c** Overgrowth of the femoral neck (8.5 mm) was visible 8 years following injury

Twelve patients (40 %) had femoral neck overgrowth (Figs. 2 and 3) as seen in the radiographs. The average overgrowth at the last follow-up was 6.2 mm (range 3.2–8.5 mm). Five patients (17 %) had an average of 6.6 mm of femoral neck shortening (range 4.2–10.2 mm), and the other 13 patients (43 %) had a femoral neck length discrepancy within 3 mm compared to the uninjured hips. The mean LLD was 0.15 cm (range 0–0.5 cm) in the overgrowth group and 0.32 cm (range 0–1.2 cm) in the non-overgrowth group. None of the patients showed severe LLD of more than 2 cm.

We categorized the patients into an overgrowth group (12 patients, 40 %) and non-overgrowth group (18 patients, 60 %). We further analyzed parameters between the overgrowth group and the non-overgrowth group (Table 1). The patients in the overgrowth group were younger than those in the non-overgrowth group (*p* = 0.0002). No AVN was noted in the overgrowth group; however, 11 patients (61 %) in the non-overgrowth group had AVN (*p* = 0.0006). The patients in the overgrowth group had better Ratliff’s scores than those in the non-overgrowth group (*p* = 0.0026).

**Table 1:** The risk factors affecting the overgrowth of the femoral neck between two groups

	Non-overgrowth (*n* = 18)	Overgrowth (*n* = 12)	*p* value
Mean age (years) (range)	9.9 (5 to 12)	5.5 (1.5 to 10)	0.0002*
Gender			0.4611
Male	13	7	
Female	5	5	
Delbet type			0.9848
I, *n* (%)	3 (17)	2 (17)	
II, *n* (%)	10 (55)	7 (58)	
III, *n* (%)	5 (28)	3 (25)	
CRIF, *n* (%)	17 (94.4)	12 (100)	1.0000
AVN, *n* (%)	11 (61.1)	0 (100)	0.0006*
Premature physeal closure, *n* (%)	6 (33.3)	0 (100)	0.0568
Coxa vara	3 (16.7)	0 (100)	0.2551
Coxa valga	1 (5.5)	1 (8.3)	1.0000
Ratliff’s score			0.0026*
Good, *n* (%)	5 (27.8)	11 (91.7)	
Fair, *n* (%)	10 (55.8)	1 (8.3)	
Poor, *n* (%)	3 (16.6)	0 (0)	

*CRIF* close reduction and internal fixation, *AVN* avascular necrosis of the femoral head

*A *p* value of <0.05 was considered to be statistically significant

## Discussion

The first report related to the lengthening of femoral neck was in 1986. Leung and Lam reported the incidence of the lengthening of femoral neck was 15 % (6 of 41) after a long-term follow-up of children after femoral neck fracture [7]. The lengthening of femoral neck ranged from less than 1 cm to more than 2 cm in their series. They reported a patient with coxa vara and long femoral neck after the 15-year follow-up. Accordingly, an increase in length of femoral neck was not just related to coxa valga. Misunderstanding valgus orientation as the true lengthening of the femoral neck may be a concern. In our study, each group had one patient who developed coxa valga in the last follow-up (*p* = 0.2551, Table 1). Therefore, we thought coxa valga did not affect the measurement of femoral neck length in our study.

In our study, overgrowth of the femoral neck was visible by 12 months post-injury in 12 patients (40 %). The average overgrowth length was 6.2 mm (range 3.2–8.5 mm) after a mean follow-up of 4.9 years. Young-age patients without complications were the factors for overgrowth of femoral neck following hip fractures. All patients with overgrowth had no related symptoms and better functional outcomes. It spoke to the impact of this phenomenon that overgrowth of femoral neck following hip fractures in children was a predictor of good outcomes.

Accumulating evidence has demonstrated femoral shaft overgrowth after femoral shaft fractures in children, and the factors affecting this overgrowth include the location of the fracture [2, 15, 16], fracture type [17], and an age from 2 to 10 years [15]. We speculate that overgrowth of the femoral neck following hip fracture is the same as femoral shaft overgrowth following femoral shaft fractures. However, the exact cause of this phenomenon is still unknown. Severe trauma has been reported to disrupt blood supply to the growth plate and the femoral neck which affects the healing of femoral neck fractures [9]. The subsequent complication of osteonecrosis of the femoral head would also affect neck length because of the vascular insult. Finally, younger age was also a factor affecting the femoral neck overgrowth during the healing period. But more clinical studies are needed to support this phenomenon.

The limitations of this study include the low power (limited number of patients) and the reliability of measurement accuracy. Skeletal measurements based on radiographs, including dual-energy X-ray absorptiometry, are subject to significant errors (magnification by about 12 % in plain radiographs [18] versus 7 % in dual-energy X-ray absorptiometry imaging [19, 20]). An error of 0.9 mm has been reported for computed tomography compared to 2.4 mm for standard radiographs [21]. Standard radiographs (placing the thighs and toes together with the feet at 15°~30° internal rotation) cannot always be achieved because of patient-related factors. According to Michelotti’s [13] observation, the hips lie in external rotation on injury film because the patients are more comfortable in that position. Therefore, we defined 3-mm lengthening as a significant overgrowth of the femoral neck. Besides, the interobserver reliability was excellent for the femoral neck length measurement. Finally, this is an observational and retrospective study, and further studies are needed to validate our findings.

## Conclusions

The rate of femoral neck overgrowth after pediatric hip fractures was 40 % in this study, with an average overgrowth of 6.2 mm (range 3.2–8.5 mm). The overgrowth phenomenon of the femoral neck did not influence poorly the functional results; instead, it was a predictor of good outcomes in the pediatric patients following hip fractures. However, further prospective study is necessary to observe the effect of the overgrowth on the clinical outcomes.

## Abbreviations

AVN: avascular necrosis of the femoral head
CRIF: close reduction and internal fixation
ICC: intraclass correlation coefficient
SPSS: Statistical Package for Social Sciences
